# Deciphering DNA methylation signatures of pancreatic cancer and pancreatitis

**DOI:** 10.1186/s13148-019-0728-8

**Published:** 2019-09-06

**Authors:** Francesco Natale, Maria Vivo, Geppino Falco, Tiziana Angrisano

**Affiliations:** 1 Department of Biology, University of Naples Federico II, 80126 Naples, Italy; 2 Biogem Scarl, Istituto di Ricerche Genetiche “Gaetano Salvatore”, 83031 Ariano Irpino, Italy

**Keywords:** Chronic pancreatitis, Pancreatic cancer, Cell-free DNA, DNA methylation, Diagnostic methods, Pre-neoplastic lesions

## Abstract

**Background:**

Chronic pancreatitis presents a high risk of inflammation-related progression to pancreatic cancer. Pancreatic cancer is the fourth leading cause of cancer-related death worldwide. The high mortality rate is directly related to the difficulty in promptly diagnosing the disease, which often presents as overt and advanced. Hence, early diagnosis for pancreatic cancer becomes crucial, propelling research into the molecular and epigenetic landscape of the disease.

**Main body:**

Recent studies have shown that cell-free DNA methylation profiles from inflammatory diseases or cancer can vary, thus opening a new venue for the development of biomarkers for early diagnosis. In particular, cell-free DNA methylation could be employed in the identification of pre-neoplastic signatures in individuals with suspected pancreatic conditions, representing a specific and non-invasive method of early diagnosis of pancreatic cancer. In this review, we describe the molecular determinants of pancreatic cancer and how these are related to chronic pancreatitis. We will then present an overview of differential methylated genes in the two conditions, highlighting their diagnostic or prognostic potential.

**Conclusion:**

Exploiting the relation between abnormally methylated cell-free DNA and pre-neoplastic lesions or chronic pancreatitis may become a game-changing approach for the development of tools for the early diagnosis of pancreatic cancer.

**Electronic supplementary material:**

The online version of this article (10.1186/s13148-019-0728-8) contains supplementary material, which is available to authorized users.

## Background

Chronic pancreatitis (CP) represents a spectrum of persisting fibro-inflammatory disorders of the exocrine pancreas that alter the organ’s typical structure and functions, and significantly reduce patients’ quality of life [1]. Genetic predisposition [2, 3], neoplasms, intraductal obstruction, or autoimmune pancreatitis (AIP) can all cause CP [4]. Alcohol history is considered a significant risk factor, and tobacco smoking may act synergistically with alcohol [5]. CP affects ∼ 50 per 100,000 individuals worldwide, with an incidence that is expected to increase over time [5]. The disease often presents with upper abdominal pain, nausea or vomiting, and steatorrhea. The diagnosis is based on tests on pancreatic function or structure, such as elevated amylase or lipase serum levels, secretin stimulation, or computed tomography.

The persistent inflammatory state in CP promotes accelerated tissue repair, which may result in neoplastic (trans)formation. For this reason, CP represents one of the highest risk factors for the development of pancreatic tumors (PTs) [6, 7]. PT is an aggressive disease usually asymptomatic at an early stage displaying symptoms resembling CP once it is overt [8]. This feature hinders early diagnosis, contributing to high observed mortality (5-year survival: ∼ 6%) [9, 10]. The global incidence rate of PT in 2012 was about six per 100,000 individuals [9] and, coherently with CP, it is expected to increase over time.

In the absence of an underlying PT, the lag period between CP diagnosis and tumor is usually one or two decades [11, 12]. This long latency period might offer a strategic opportunity for early diagnosis and curative treatment once biomarkers with robust predictive power are discovered.

The best-known biomarker for PT is the serum protein-carbohydrate antigen 19-9 (CA19-9, or sialylated Lewis antigen) [13]. When released by a PT, CA19-9 levels can help to monitor the treatment or the relapse of the disease [14]. Unfortunately, elevated CA19-9 levels are also present in CP or other cancers [15]. Besides, about 10% of the Caucasian population lacks CA19-9 on their red blood cells [15]. Due to these limitations, the American Society of Clinical Oncology discouraged the use of CA19-9 as a biomarker for PT diagnosis [14]. The lack of robust non-invasive diagnostic screening methods has propelled the research of potential biomarkers in patients’ biological fluids. Sequence analysis of cell-free DNA (cfDNA) from the bloodstream of patients affected by PT uncovered mutations in the *KRAS* already in the early 90s [16], and improvement of these methods was attained in the past years [2, 17]. A more recent and emerging field investigates the methylation levels of circulating cfDNA. This approach is actively used for the discovery of biomarkers of several diseases [18–20]. Its popularity stems from the increasing evidence that cfDNA carries methylation marks that enable the identification of tissue-specific cell death [21] and are more broadly informative, sensitive, and specific than individual DNA mutations [22–24]. Further, sample collection is minimally invasive and allows adequate follow-up under negligible stress conditions for the patient.

In this review, we provide an overview of the molecular determinants of PT and the genes showing differential DNA methylation in CP, pre-neoplastic lesions of the pancreas, and PT. In particular, we will compare studies conducted either on primary tissue biopsies or from biological fluids. We aim to decipher disease-specific methylation patterns in pancreatic diseases to serve as a novel diagnostic or prognostic tool for PT. The identification of a “pancreatic disease signature” to distinguish between inflammation and cancer could hopefully enhance non-invasive tools for the early diagnosis of PTs.

## The molecular landscape of pancreatic cancer and pancreatitis

The pancreatic adenocarcinoma develops almost exclusively from the exocrine pancreatic ductal epithelium cells accounting for 85% of all PTs. Other PTs include the acinar cell carcinoma originating from the exocrine acini of the pancreas, the pancreatic neuroendocrine tumors (PNETs) arising from neuroendocrine cells, and other minorities. For its abundance, we will hereafter use the term PT to refer to pancreatic ductal adenocarcinoma, unless otherwise specified.

**Fig. 1:**
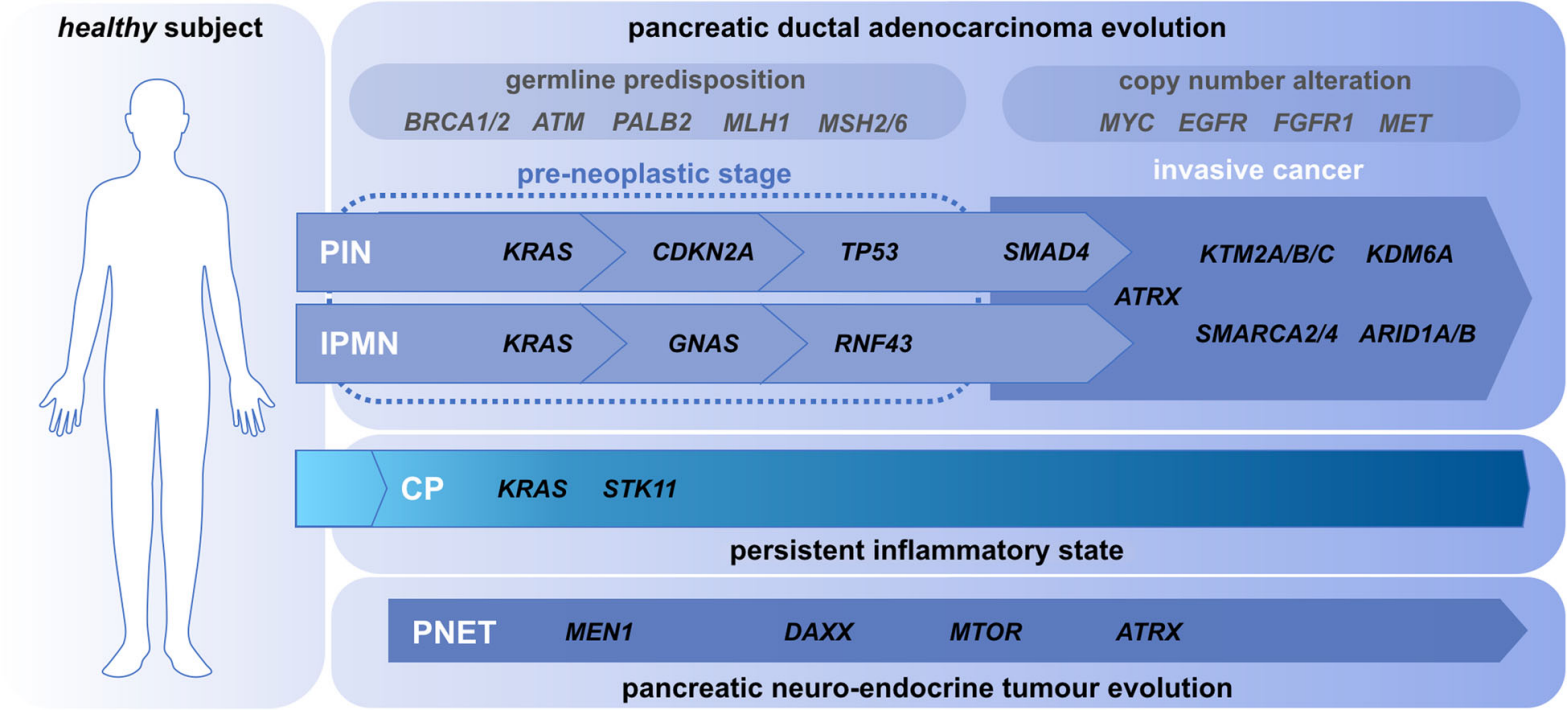
Genetic landscape of pancreatic cancer. Genes mutated in pancreatic ductal adenocarcinoma (PT, top) and pancreatic neuroendocrine tumor (PNET, bottom). For the former, genetic predisposition or copy number alterations are also reported (reviewed in [25]). At the pre-neoplastic stage (dotted line), pancreatic intraepithelial neoplasias (PIN) and intraductal papillary mucinous neoplasms (IPMN) coexist. Overall, aberrant homeostasis of genes regulating cell differentiation, proliferation and apoptosis promote the transition from pre-neoplastic to advanced stages of the disease [26–31]. Chronic pancreatitis (CP) may also be present, contributing to the progression of an underlying tumor

Molecularly, a variety of genetic and epigenetic events underlie the development of PT (Fig. 1).

Pre-neoplastic non-invasive lesions are thought to be the first stage of PT etiopathogenesis. Low-grade pancreatic intraepithelial neoplasia (PIN) is the most frequent microscopic lesion of the pancreas, harboring mutations in the *KRAS* in about 90% of the cases [8, 32]. As the grade of the lesion progresses, additional mutations in cell cycle-regulating *CDKN2A* and *TP53* are found [8]. Intraductal papillary mucinous neoplasms (IPMNs) are macroscopic lesions and have a 25% risk of developing into invasive PT. Other than *KRAS*, they often present mutations in genes involved in the Wnt signalling pathway (e.g., *GNAS* and *RNF43*) [8]. Other types of lesions include mucinous cystic neoplasms and intraductal tubulopapillary neoplasms [32, 33].

Genes mutated in PNETs are generally different from those found in exocrine PTs [34]. For example, *KRAS* mutation is absent in most PNETs, while frequent mutations in these tumors occur in *DAXX* (encoding the transcriptional corepressor death domain-associated protein 6), *MTOR* (encoding the mammalian target of rapamycin kinase), and *MEN1* genes [35].

At the invasive stage, PTs present mutations in *SMAD4* (about 55% of the cases) and genes of the SWI/SNF chromatin remodeling family (∼ 15% of the cases) [6, 8]. A member of this family, ATRX, is frequently mutated in PNETs [35], and these events have been shown to cause changes in DNA methylation patterns [36, 37]. The latter, together with aberrant DNA hydroxymethylation, are established processes contributing to cancer development [38–41].

Inflammatory stimuli may lead to aberrant DNA methylation homeostasis and, coherently, to gene expression changes [39, 42–44]. Further, the progressive increase of DNA methylation levels has been described in chronic inflammatory diseases developing into cancer. The interactions between inflammation and epigenetics in tumor initiation, promotion, and immune evasion can be leveraged in cancer prevention and treatment [40]. Thus, it is not surprising that the relative risk for CP patients developing PT is generally > 10 [11, 45]. In this scenario, chronic inflammation may inactivate the oncogene-induced senescence barrier that is typical for pre-neoplastic PIN lesions and, thus, it promotes the neoplastic progression of PT [46]. Genetically, 4–28% of CP cases show KRAS mutation [2, 3].

Conversely, no *KRAS* mutation at known tumor-promoting sites has so far been observed in AIP [47]. Finally, the mutation of the *STK11*, a known risk factor for PTs, has recently been described in CP [17]. In the past two decades, a significant number of studies conducted in pancreatic cell lines, xenografts, or primary tissue specimens aimed at identifying aberrant DNA hyper- or hypomethylation targets in PTs. Almost all DNA methylation analyses rely on PCR-based methods, on bisulfite-treated—or not—specimens [48]. For their high throughput, microarray or—more recently—next-generation sequencing (NGS) methods enabled the discovery of many target genes. The fast and cost-containing methylation-specific PCR (MSP) is generally the method of choice for target validation. This approach led to the identification of many candidate genes associated with epigenetic changes taking place during the carcinogenesis of PT. In this section, we will discuss those genes whose DNA methylation levels hold potential for the differential diagnosis of CP, pre-neoplastic conditions of the pancreas, and invasive PTs. Besides, the genes, as well as the method employed for their discovery and validation, will be described.

## Differentially DNA-methylated genes in pancreatic tumor and pancreatitis

The first studies comparing cancerous and healthy pancreatic tissues analyzed the methylated status of known tumor-suppressors and cancer-associated genes. Also, specific CpG islands spread within the genome were found preferentially methylated in tumors (MINT loci). In particular, the DNA methylation status of CpG islands embedded in several gene promoters (*CACNA1G*, *CDH1*, *CDKN2A*, *DAPK1*, *MGMT*, *MINT1*-*2*-*31*-*32*, *MLH1*, *RARB*, *THBS1*, and *TIMP3*) was analyzed in PT xenografts [49, 50]. All cancerous specimens showed aberrant DNA methylation of at least one locus except for *MGMT*, which was non-methylated in either neoplastic or normal samples. Simultaneous methylation of at least four loci was a feature of ∼ 14% of PT xenografts. Overall, the most frequently methylated loci were *MINT32*, *MINT1*, and *MINT28*. Moreover, seven CpG islands (CGIs), of which three associated to the known *CCNG1*, *PENK*, *ZBP* genes, were found differentially methylated in pancreatic-derived cell lines compared with the healthy pancreas and validated in PTs [51, 52]. Among these *PENK*, CGI was found methylated in 91% of cases. Of note, five specimens of CP were also analyzed, showing *PENK* CGI methylation in two cases [52].

*PENK*, as well as *CDKN2A*, DNA methylation status was further investigated in PINs of different grade: intraductal PTs, extra-ductal PTs (including one PNET), and CP specimens [53, 54]. *PENK* methylation was present in 93% of invasive PTs, of which 27% presented *CDKN2A* methylated. In contrast, non-neoplastic specimens resected from matched healthy tissues did not harbor methylation of either gene. Noteworthy, the prevalence of *PENK* methylation increased significantly with increasing lesion grade (from 8 to 46%, in grade 1A or grade 3 lesions, respectively). Only one of the extra-ductal PTs presented *CDKN2A* methylation, while CP specimens had neither gene methylated. Interestingly, as regarding *CDKN2A*, available data suggest that hypermethylation associated with the loss of *CDKN2A* expression might occur only in CP in which low-grade PINs were observed [45]. In line with its broad role as a tumor suppressor, the downregulation of *CDKN2A* expression was found in this study independently of methylation status.

These data support the disease stage-specific DNA methylation model and qualify these genes as potential biomarkers of early pancreatic carcinogenesis.

Comparing invasive PTs to IPMNs, 6% of IPMNs and 22% of invasive PTs presented *SOCS1* methylation. In contrast, none of the pancreatic normal ductal epithelia and the PINs that were analyzed presented *SOCS1* methylation [55]. In a preliminary study, the analysis of IPMNs with different grades of invasiveness reported SOCS1 methylation in 6% of invasive IPMNs [56], providing evidence that, therefore, this gene might be an early indicator of invasiveness of the disease. About half of the tested specimens scored positive for *CDKN2A* (*p16INK4a* specific region) and *TP73* methylation, independently of their grade of invasiveness. Conversely, *CDKN2B*, *ESR1*, and *TIMP3* were methylated in none of the examined specimens. More frequent methylation of *APC* (50% vs. 10%), *CDH1* (38% vs. 10%), *MGMT* (45% vs. 20%), and *MLH1* (38% vs. 10%) was observed in invasive IPMNs, compared to non-invasive samples. Unfortunately, the weakness of these findings results from the limiting size of the non-invasive IPMN subpopulations [45].

A progressive increase of the DNA methylation of at least one gene and the average number of methylated genes was observed from PIN to PT in 58 patients who had undergone resection surgery for invasive PTs.

The impact of the inflammatory environment on DNA methylation was assessed. The data showed that DNA methylation also increases with inflammation of pancreatic tissue. *BRCA1*, *APC*, *CDKN2A*, and *TIMP3* were methylated in 60%, 59%, 39%, and 31% of the PT cases, respectively [57]. Finally, the DNA methylation levels of six genes frequently hypermethylated in PT (*CCND2*, *CDKN2A*, *FOXE1*, *NPTX2*, *PENK*, *TFPI2*) were analyzed in AIP specimens [47], showing no significant hypermethylation in both AIP or healthy pancreas.

Evidence of DNA hypomethylation events in PTs is also reported [58–60]. A panel including 18 genes known to be over-expressed in PTs and 14 genes whose overexpression in PT was not documented was composed. MSP analysis revealed methylation of 19 of 32 genes in healthy pancreas. All genes that were known to be transcribed at high levels in PTs but not expressed in healthy pancreas (*CLDN4*, *LCN2*, *MSLN*, *PSCA*, *S100A4*, *TFF2*, and *YWHAS*) were frequently hypomethylated in PT xenografts. In particular, five or six genes showed simultaneous DNA hypomethylation in 92% or 61% of the cases, respectively. Moreover, from 379 identified loci hypomethylated in PT with respect to healthy pancreas [61], the oncogenes *FOS*, *JUNB*, and *MYB*; the genes *NDN* and *SMARCA1* [62]; and the chromatin modifiers genes such as *CTR9*, *EP400*, *HIRIP3*, *KDM6A*, *KMT5A*, and *PRMT1* were identified. These genes presented increased expression in PT specimens playing a role in core signalling pathways of PT [60].

In Additional file 1: Table S1, we reported a list of several studies that analysed the methylation status of different genes. Genes, commonly addressed in both primary tissues and liquid biopsies (see next section), were grouped and highlighted in gray.

### Genome-wide profiling of methylated genes in pancreatic tumor and pancreatitis

The advent of genomic technologies improved the screening capability by several orders of magnitude, providing promising results. Using a high-throughput microarray approach, 11 genes (*CDH3, CLDN5, FOXE1, LHX1, NPTX2, RPRM, SFRP1, ST14, TJP2, UCHL1*, and *WNT7A*) markedly induced after treatment with 5-aza-2-deoxycytidine (5Aza-dC), a DNA demethylation agent, were specifically identified in pancreatic tumor cell lines [63]. Analysis of the methylation status of these genes in PTs showed that four genes (*CLDN5*, *NPTX2*, *SFRP1*, *UCHL1*) were methylated in at least 93% of the screened specimens, while only two (*CDH3* and *ST14*) presented methylation in less than 20% of samples.

A microarray-based method was used to interrogate 27,800 CGIs covering 21 Megabase-pairs of the human genome. Then, 1968 CGIs showed differential methylation in pancreatic cancer cell lines compared to a healthy pancreas. Validation in 57 PTs and 34 normal pancreases confirmed specific methylation of *MDFI*, *MIR9*-*1*, *ZNF415*, *CNTNAP2*, and *ELOVL4* in cancer-derived samples [64].

Further application of this strategy resulted in the identification of > 1200 known loci that presenting specific methylation in PT. Many of these loci belonged to Wnt signalling pathway or the homeobox and the cadherin superfamilies [61]. Analysis of 24 of these hypermethylated candidate genes (*ADCY5*, *BMI1*, *BTBD6*, *CACNA1H*, *EFNA4*, *FOXF2*-*G1*, *FZD1*-*2*-*7*, *HIC1*, *ID4*, *IRF5*, *NEUROG3*, *PCDH17*, *PDE4B*, *SFRP1*, *SIM1*, *SMOC2*, *SOX3*-*15*, *WNT3*-*5A*, *ZBTB16*) was validated in PT showing that for 20 genes DNA methylation was, on average, 50% more frequent in PTs. Only for *EFNA4*, *BTBD6*, and *FZD2*-*7* DNA methylation levels were comparable between the two conditions [61]. A transcriptomics-based screening of pancreatic cell lines identified—from a pool of > 1400 genes—eight potential biomarkers showing PT-specific DNA methylation signatures [65]. This gene-panel was then used to probe a large cohort of PT, CP, and healthy pancreas specimens. The most frequently methylated genes in PTs were *BNC1* and *ADAMTS1*. Quantitative MSP (qMSP) validation confirmed the results. Interestingly, *BNC1* methylation was detected in PINs, while *ADAMTS1* methylation was exclusively found in invasive PTs. Importantly, these two genes showed increased DNA methylation in pre-neoplastic PINs, with little to no DNA methylation in CP or healthy pancreas [65], thus underlining their potential use for early PT diagnosis and prognosis. In a comprehensive program, composed of four case-control studies, PT-specific DNA-methylated markers were further identified through NGS [22]. Analysis of > 1,000,000 CpG sites derived from matched PTs, benign pancreas, and healthy colon tissue specimens resulted in the identification of > 500 DNA-methylated regions (DMRs). Interestingly, upon biological validation, six of 87 candidate genes (*CD1D*, *CLEC11A*, *IKZF1*, *KCNK12*, *NDRG4*, *PRKCB*) were further selected and tested as diagnostic biomarkers in cfDNA specimens (as described in next section) [22].

NGS was also utilized to identify differentially DMRs in healthy and neoplastic samples. Aberrantly methylated CGIs were more frequent in PT, compared to the healthy pancreas, and DNA hypermethylation events in PTs typically occurred in the vicinity of the transcription start site (TSS). Several individual DMRs (including *C5orf38*, *DLX4*, *ELAVL2*, *EMX1*, *IRX1*, *NPR3*, *PITX2*, *SIM2*, *TBX5*, *TFAP2C*, and *VSTM2B*) were further validated by target-specific methods (MSP, direct bisulfite sequencing or methylation-sensitive restriction endonuclease PCR) [58]. It is well-known that DNA methylation can occur in diverse genomic contexts such as promoters, CGIs and CGI shores, introns, exons, and miRNAs. Epigenetic deregulation of non-coding genes like microRNAs (miRs) is an established event in tumor development. miRNA are commonly involved in inflammatory processes playing a role in coordinating several features of the immune system, including immune cell differentiation, function, and recruitment.

Three *MIR124* genes and *MIR10B* showed a higher mean CpG methylation fraction in PTs compared with matched non-cancerous tissues. Hypermethylation induced the silencing of *MIR124*, which was associated with poor prognosis. The functional implications of such findings derive from *MIR124* role in inhibiting cell proliferation, invasion, and metastasis. Among *MIR124* direct targets, stem Rac1, a putative PT-promoting factor that activates JNK-dependent cell motility [66–70].

Also, *MIR210* and *MIR130B* displayed PT-specific DNA-hypomethylation. Supporting evidence relates to high levels of *MIR210* to PT progression, epithelial-to-mesenchymal transition, and adverse prognosis in cancer patients [71, 72]. For *MIR130B*, its DNA methylation levels [73] and the relative functional implications are still controversial: while expression of *MIR130B* is reported to inhibit cell proliferation and invasion in PTs by direct targeting of STAT3 [74], increased levels of this miR associates with the development of other neoplastic diseases [75].

## Pancreatic cancer and pancreatitis cfDNA methylation signatures in biological fluids

To find a diagnostic or prognostic tool relevant for PT, several studies, rather than analyzing only one gene, combined many candidate genes into aggregate biomarkers. The methylation levels of the different genes in the aggregate were adopted to differentially diagnose pre-neoplastic conditions or PT from different biological fluids, such as pancreatic juice and blood, as indicated in Table 1.

**Table 1:** List of cfDNA methylation aggregate biomarkers with high sensitivity for chronic pancreatitis or pancreatic cancer diagnosis

Aggregate biomarkers		Comparison	Diagnostic method	Test performance (95% CI)		Reference
				Combined sensitivity	Combined specificity	
*CLDN5*	*NPTX2*	CP vs. PT	MSP	75%	75%	Sato et al. 2003a [59]
*SFRP1*						
*CCND2*	*FOXE1*	N + CP vs. PT	qMSP	82%	100%	Matsubayashi et al. 2006 [76]
*NPTX2*	*PENK*					
*TFPI2*						
*CCND2*	*PLAU*	N vs. PT	MCAM	76%	59%	Melnikov et al. 2009 [77]
*SOCS1*	*THBS1*					
*VHL*						
*BRCA1*	*CCND2*	N vs. CP	MCAM	82%	78%	Ligget et al. 2010 [80]
*CDKN1C*	*MLH1*					
*PGR* (distal)	*PGR* (proximal)					
*SYK*	*VHL*					
*CDKN1C*	*CDKN1B*	CP vs. PT	MCAM	91%	91%	Ligget et al. 2010 [80]
*CCND2*	*DAPK1*					
*ESR1(A)*	*MGMT*					
*MLH1*	*MUC2*					
*MYOD1*	*PGK1*					
*PGR* (proximal)	*RARB*					
*RB1*	*SYK*					
*ADAMTS1*	*BNC1*	N vs. PT	qPCR	81% (69%–93%)	85% (71%–99%)	Yi et al. 2013 [65]
*APC*	*BMP3*	CP vs. PT	MSP	76%	83%	Henriksen et al. 2016 [82]
*BNC1*	*MESTv2*					
*RASSF1A*	*SFRP1*					
*SFRP2*	*TFPI2*					
Age (> 65)						

*CP* chronic pancreatitis, *PT* pancreatic adenocarcinoma, *N* normal. The gene names’ was included in gene names’ abbreviation section

While in PT solid biopsies, six methylated genes were identified to be predictive of advanced disease; in pancreatic juice, only three of them (*CLDN5, NPTX2*, and *SFRP1*) showed the potential to be employed as aggregate biomarkers [63]. Indeed, aberrant DNA methylation of at least one of the three was detectable in 75% of the pancreatic juice specimens from PT and in none of the benign samples. The absence of DNA methylation for all three genes was observed in 16% of PTs, resulting in a specificity of 75%. Of note, four PNETs were also probed and displayed no DNA methylation. These results were encouraging, and pancreatic juice from larger cohorts was examined in other studies.

For example, Matsubayashi et al. investigated pancreatic juice endoscopically or surgically collected from individuals with suspected pancreatic diseases, including CP and benign pancreatic lesions [76]. Quantitative interrogation of 17 target genes revealed increased DNA methylation levels in all PT specimens (*n* = 56), compared to the normal pancreas (*n* = 11). Further, the number of methylated genes in the high-risk PT group (*n* = 44) was more abundant than in healthy controls but similar to patients with chronic pancreatitis (*n* = 11). Combination of the five most discriminating assays (*CCND2*, *FOXE1*, *NPTX2*, *PENK*, and *TFPI2*) scoring DNA methylation in more than one gene was highly predictive of PT: nine of 11 patients with PT but none of the 64 individuals without detectable pancreatic neoplasia presented DNA methylation signal (82% sensitivity and 100% specificity for PT). Interestingly, these genes were previously identified in several studies conducted on pancreatic biopsies. Although none of the target genes was useful to distinguish CP from healthy pancreases, this aggregate biomarker successfully discriminated PTs from other pancreatic lesions.

In their clinical pilot testing, Kisiel et al. assessed the DNA methylation levels of six previously identified candidate biomarkers, together with mutant *KRAS* status, on secretin-stimulated pancreatic juice samples from 61 PT patients, 22 CP patients, and 19 with healthy pancreas [22]. In line with previous findings, mutant *KRAS* had a sensitivity of 56% and 39% (at 90% specificity) in discriminating PTs or CP from healthy pancreases, respectively. Overall, the other biomarkers performed better: the sensitivity (at 90% specificity) in discriminating PT from healthy pancreas was 79% for *CD1D*, 67% for *CLEC11A*, 62% for *IKZF1*, 79% for *KCNK12*, 72% for *NDRG4*, and 67% for *PKRCB*. The sensitivity (at 90% specificity) in discriminating PT from CP was generally lower: 53% for *CLEC11A*, 54% for *IKZF1*, 46% for *KCNK12*, and 67% for *NDRG4*. Of note, *CD1D* displayed a better discriminating potential between PT and CP (84%), while PKRCB methylation was worse (38%) than mutant *KRAS* at distinguishing PTs from CPs.

The collection of pancreatic juice is a relatively complex procedure, and it might not be appropriate as a standard approach for early detection in asymptomatic individuals. For this, the analysis of cfDNA in plasma specimens received increasing inputs.

Melnikov et al. examined plasma specimens of PT patients with a panel of 56 frequently methylated genes [77]. By mean of a microarray-based approach [78], five promoters were selected (*CCND2*, *PLAU*, *SOCS1*, *THBS1*, and *VHL*) and combined in an aggregate biomarker. This test assesses DNA hypomethylation events, which makes comparison with techniques that detect cancer-related hypermethylation difficult. The assay distinguished healthy pancreas from PTs with 76% sensitivity and 59% specificity. Previous findings had reported *CCND2*, *SOCS1*, and *THBS1* hypermethylation as informative for cancer detection in pancreatic juice or tissues [52, 55, 79].

In a follow-up study by Liggett et al., 17 gene promoters were examined to identify differential cfDNA methylation in PT and CP patients [80]. Eight genes (*BRCA1*, *CCND2*, *CDKN1C*, *MLH1*, proximal and distal *PGR* promoter regions, *SYK*, and *VHL*) were useful to distinguish healthy pancreas from CP, with a sensitivity and a specificity of 82% and 78%, respectively. For all genes in this panel, promoter DNA methylation was more frequent in individuals with CP. Fourteen genes (*CCND2*, *CDKN1C*, *CDKN2B*, *DAPK1*, promoter A of *ESR1*, *MGMT*, *MLH1*, *MUC2*, *MYOD1*, *PGK1*, the proximal region of the *PGR* promoter, *RARB, RB1*, and *SYK*) were able to distinguish CP from PTs with a sensitivity and a specificity of 91%. It is worth noting that all genes that were hypermethylated in CP displayed hypomethylation in PT. As CP often precedes PT, dynamic DNA methylation patterns for a given set of genes might underlie the progression of the disease. Validation in larger independent cohorts would be critical to confirm these findings.

Park et al. investigated the DNA methylation levels of six genes (*CDKN2A*, *NPTX2*, *PENK*, *SFRP1*, *RASSF1A*, and *UCHL1*) in the plasma of individuals with PT and CP [81]. The investigated genes had previously been reported to present high DNA methylation in 81% of PTs (13 of 16 cases), 61% of CP (eight of 13 cases), with more than one gene affected in either condition. In contrast, less than 4% of healthy pancreases (one of 29) presented DNA methylation. In this study, high interindividual variability was observed, and significant differences between PT and CP could not be confirmed, except for *CDKN2A*, specifically methylated in PT but not in CP.

Scarcely abundant cfDNA is often a limiting factor for many studies. To overcome such issue and improve the sensitivity of DNA methylation detection, Yi et al. employed a single-tube high-yield collection method, termed methylation on beads (MOB) [65]. Based on their previous screening, the authors determined the sensitivity assuming that all patients with PT (stages I–IV; *n* = 42) would harbor *BNC1* and *ADAMTS1* gene methylation while healthy subjects (*n* = 26) would not [65]. Further, 33 of 42 PT patients showed *BNC1* methylation, while 20 of 42 showed *ADAMTS1* methylation, with a sensitivity of 79% and 48% for *BNC1* and *ADAMTS1*, respectively. Specificity was 89% for *BNC1* and 92% for *ADAMTS1*. Combining both genes slightly improved the overall sensitivity (81%) but worsened the overall specificity (85%).

A recent prospective study from Henriksen et al. employed a panel of 28 genes to assess the DNA methylation state in patients with PT (*n* = 95), CP (*n* = 124), and acute pancreatitis (*n* = 59) from plasma specimens [82]. Compared with CP, patients with PT presented more methylated genes, on average (8.4 vs. 4.7). The authors developed a diagnostic prediction model that optimized the combination of biomarkers to achieve the highest predictive power. The model included eight genes (*APC*, *BMP3*, *BNC1*, *MESTv2*, *RASSF1A*, *TFPI2*, and *SFRP1*-*2*) and patient’s age (> 65). Although not quantitative, this model successfully distinguished malign from benign conditions with a sensitivity of 76% and a specificity of 83%, independently of the tumor stage.

Finally, a recent study by Lehmann-Werman et al. revealed how cfDNA methylation patterns could be employed to detect tissue-specific cell death in individuals affected by PT or CP [21]. Array-based methylome data from cadaveric material of different organs showed that *CUX2* and *REG1A* could be employed as pancreas-specific markers. While the former was preferentially unmethylated in ductal pancreatic cells, the latter was found unmethylated in both acinar and ductal pancreatic cells. cfDNA from matched plasma specimens showed high levels of *CUX2* and *REG1A* in individuals with late-stage PT or CP. Levels of these markers were lower as the grade of the lesion decreased, and it was minimum in healthy individuals, leading to the conclusion that tissue-specific cfDNA levels in the plasma directly reflect cell death, irrespective of the etiology of the disease. Besides, individuals affected by PT presented high levels of the ductal-specific marker *CUX2*, whereas those affected by CP had higher levels of the acinar-ductal marker *REG1A*. In summary, hypomethylation of exocrine pancreas-specific cfDNA is detected in the blood of patients with PT and CP, mirroring the death of exocrine cells in these conditions.

## cfDNA methylation as a diagnostic marker of pre-neoplastic lesions

Liquid biopsies are convenient and minimally invasive if compared to the more cumbersome and challenging procedure of tissue sampling. However, their utilization for the development of cfDNA methylation biomarkers presents some pitfalls. Despite the high tissue specificity of cfDNA methylation patterns [21] and the introduction of high-yield collection methods [65], the scarce abundance of disease-specific cfDNA remains a limiting factor. Thus, to achieve robust data analysis, the investigation has to be restricted to a few targets.

At present, what appears to be clear is that single epigenetic markers are seldom sufficient to distinguish pancreatic malign from benign or pre-neoplastic conditions. The simultaneous evaluation of more biomarkers has thus been investigated through systematic approaches. Today, prediction models are extensively used in a variety of biological fields [83, 84], and models integrating the DNA methylation status of target genes have been developed to provide prognostic estimates for individuals with PT [85]. Similarly, prediction models enabled the development of optimized aggregate biomarkers that combined different disease-specific informative targets, achieving high predictive power [82]. These models successfully distinguished PT from non-neoplastic conditions or CP with encouraging sensitivity and specificity. Besides, the utilization of stratification (e.g., age) or prognostic parameters may be a valid strategy to improve sensitivity. Recent evidence indicates that aberrant DNA methylation events correlate with longer patients’ survival [73]. As can be seen from Table 1, which shows the aggregate biomarkers discussed in this review, the grouped genes analyzed by Ligget’s coworkers represent an excellent combination of sensitivity and specificity [80].

**Fig. 2:**
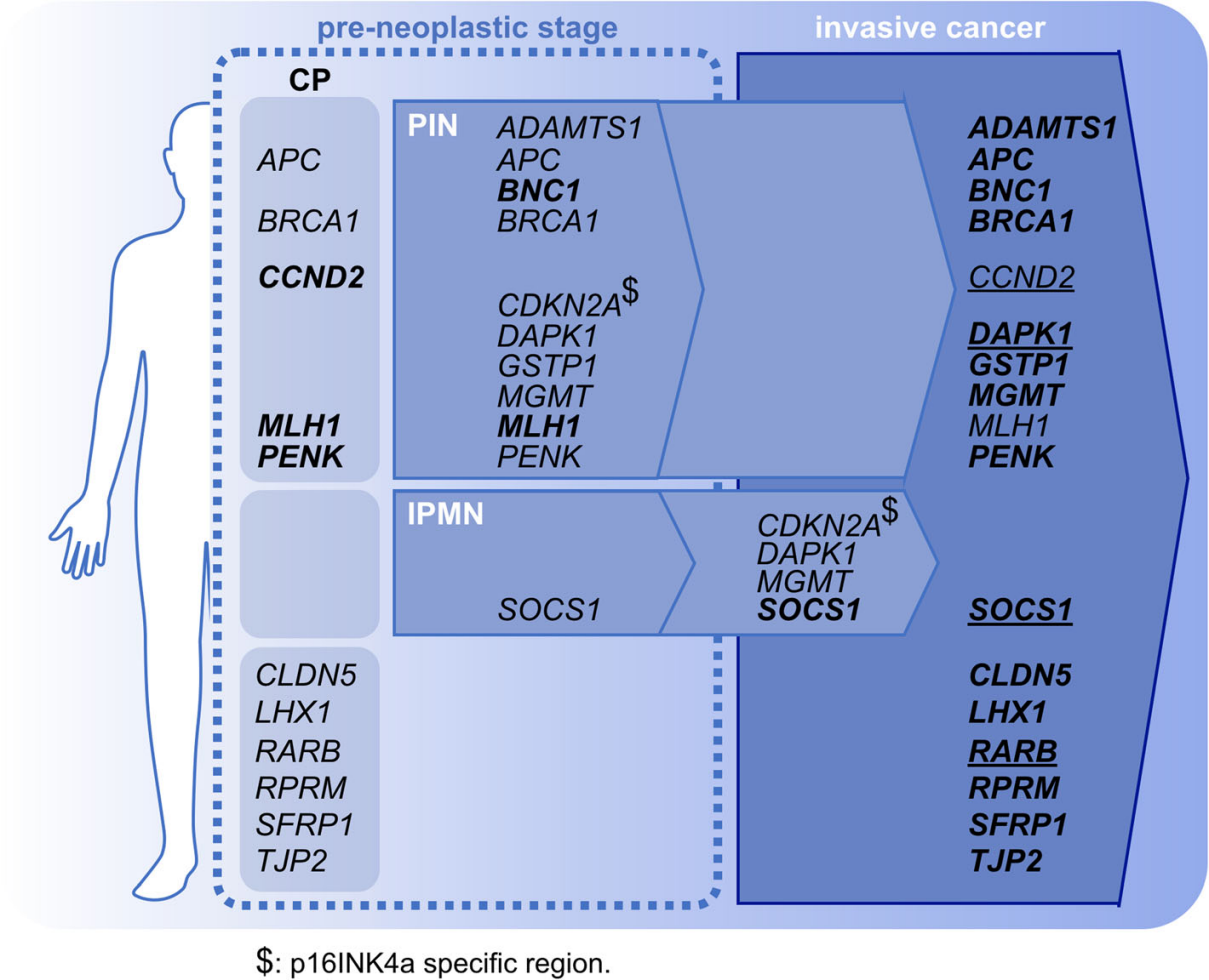
Epigenetic landscape of pancreatic cancer. Graphical representation of the genes presenting different DNA methylation levels in CP, pre-neoplastic conditions, and invasive PT. Genes in bold present high DNA methylation levels, while normal formatting indicates that methylation is detected, though at lower levels. Underline genes present controversial results (e.g., hypermethylation is reported by different studies). $: p16INK4a specific region

Figure 2 reports a schematic representation of the genes that may provide DNA methylation stage-specific information of pre-neoplastic conditions.

For some genes, DNA methylation may indeed mirror different stages of the disease progression (e.g., *ADAMTS1* or *SOCS1*, in PINs or IPMNs, respectively) [55, 56, 65]. Other genes show dynamic DNA methylation levels that are increased by the persistent inflammatory environment (e.g., CP) [57], yet they are unmethylated at advanced stages of the disease (e.g., *CCND2*) [80]. On the other hand, genes such as *APC*, *BRCA1*, *CLDN5*, *LHX1*, *RPRM*, *SFRP1*, and *TJP2*, that progress to a state of increased methylation in invasive cancer, can be considered biomarkers of an aggressive stage of the disease [57, 63].

## Conclusions

This review aimed to focus on the present advances in liquid biopsy for PT to establish predictive aggregate biomarkers that provide benefits to patients. Many candidate genes have been discussed in this review, the majority of which are hypermethylated as the pancreatic disease progresses. Interestingly, the use of new technologies and array-based screening employed to analyze DNA methylation status indicated that hypomethylation events, rather than hypermethylation, could be more informative because their occurrence appears to be independent of the inner tumor heterogeneity [78]. Besides, the decreasing cost of next-generation sequencing technologies offers an appealing option for methodological standardization. In conclusion, early diagnosis has the potential to allow prognosis prediction, tumor-stage monitoring, and provide personalized therapeutic strategies for patients suffering from a pancreatic disease.

## Additional file

Additional file 1: **Table S1.** List of genes presenting differential DNA methylation between healthy pancreas, chronic pancreatitis or pancreatic cancer. CP: chronic pancreatitis; HiR: high PT-risk; IPMN: papillary mucinous neoplasms; PIN: pancreatic intraepithelial neoplasias; PT: pancreatic adenocarcinoma; PTX: pancreatic adenocarcinoma xenografts. Hyper:DNA hypermethylation; hypo: DNA hypomethylation; –: no change; inv.: invasive; *: non-significant; #: cohort size <10; §: p16INK4a specific region; $: p14ARF specific region; £: associated with longer survival. Grey background: genes analysed in both primary and liquid biopsies (XLS 57 kb)

## Data Availability

Not applicable.

## Abbreviations

5Aza-dC: 5-aza-2-deoxycytidine
AIP: Intraductal obstruction or autoimmune pancreatitis
CA19-9: Carbohydrate antigen 19-9
cfDNA: Cell-free DNA
CGI: CpG island
CP: Chronic pancreatitis
DMR: DNA-methylated region
DSB: DNA sequence Bisulfite
Hyper: Hypermethylation
Hypo: Hypomethylation
IPMN: Intraductal papillary mucinous neoplasm
MCA/RD: Methylation CpG island amplification/coupled with representational difference analysis
MCAM: Microarray platform
MeCap-seq: Methyl Capture sequencing
miR: MicroRNA
MSP: Methylation-specific PCR
MSREP: Methylation sensitive restriction enzyme-based qPCR
N: Normal
NGS: Next-generation sequencing
PIN: Pancreatic intraepithelial neoplasia
PNET: Pancreatic neuroendocrine tumor
PT: Pancreatic tumors
qMSP: Quantitative MSP
qPCR: Quantitative real-time PCR
RRBS: Reduced representation bisulfite sequencing
TSA: Trichostatin A
TSS: Transcription start site

## Gene names’ abbreviations

*ADAMTS1*: ADAM metallopeptidase with thrombospondin type 1 motif 1
*ADCY5*: adenylate cyclase 5
*AGAP2*: AGAP2 antisense RNA 1
*ALX4*: ALX homeobox 4
*APC*: APC regulator of WNT signaling pathway
*ASCL2*: Achaete-scute family bHLH transcription factor 2
*BMI1*: BMI1 proto-oncogene, polycomb ring finger
*BMP3*: Bone morphogenetic protein 3
*BNC1*: Basonuclin 1
*BNIP3*: BCL2 interacting protein 3
*BRCA1*: BRCA1 DNA repair associated
*BTBD6*: BTB domain containing 6
*C5orf38*: Chromosome 5 open reading frame 38
*CACNA1G*: Calcium voltage-gated channel subunit alpha1 G
*CACNA1H*: Calcium voltage-gated channel subunit alpha1 H
*CADM1*: Cell adhesion molecule 1
*CCND2*: Cyclin D2
*CCNG1*: Cyclin G1
*CD1D*: CD1d molecule
*CDH1*: Cadherin 1
*CDH3*: Cadherin 3
*CDKN1A*: Cyclin-dependent kinase inhibitor 1A
*CDKN1C*: Cyclin-dependent kinase inhibitor 1C
*CDKN2A*: Cyclin-dependent kinase inhibitor 2A
*CDKN2B*: Cyclin-dependent kinase inhibitor 2B
*CHFR*: Checkpoint with forkhead and ring finger domains
*CLDN4*: Claudin 4
*CLDN5*: Claudin 5
*CLEC11A*: C-type lectin domain containing 11A
*CNTNAP2*: Contactin-associated protein-like 2
*CTR9*: CTR9 homolog, Paf1/RNA polymerase II complex component
*CUX2*: Cut-like homeobox 2
*DAPK1*: Death-associated protein kinase 1
*DLX4*: Distal-less homeobox 4
*EFNA4*: Ephrin A4
*ELAVL2*: ELAV like RNA binding protein 2
*ELOVL4*: ELOVL fatty acid elongase 4
*EMX1*: Empty spiracles homeobox 1
*EP400*: E1A binding protein p400
*ESR1*: Estrogen receptor 1
*EVL*: Enah/Vasp-like
*EYA2*: EYA transcriptional coactivator and phosphatase 2
*FAM115A*: Family with sequence similarity 115, member C (or TCAF1: TRPM8 channel associated factor 1)
*FOS*: Fos proto-oncogene, AP-1 transcription factor subunit
*FOXE1*: Forkhead box E1
*FOXF2/G1*: Forkhead box G1 (or SNAR-G1: small NF90 (ILF3) associated RNA G1)
*FZD1*: Frizzled class receptor 1
*FZD2/7*: Frizzled class receptor 2
*GSTP1*: Glutathione S-transferase pi 1
*HIC1*: HIC ZBTB transcriptional repressor 1
*HIRIP3*: HIRA-interacting protein 3
*HPP1*: Hyperpigmentation, progressive, 1
*ID4*: Inhibitor of DNA binding 4, HLH protein
*IKZF1*: IKAROS family zinc finger 1
*IRF5*: Interferon regulatory factor 5
*IRX1*: Iroquois homeobox 1
*JUNB*: JunB proto-oncogene, AP-1 transcription factor subunit
*KCNK12*: Potassium two pore domain channel subfamily K member 12
*KDM6A*: Lysine demethylase 6A
*KMT5A*: Lysine methyltransferase 5A
*LCN2*: Lipocalin 2
*LHX1*: LIM homeobox 1
*MDFI*: MyoD family inhibitor
*MEST*: Mesoderm-specific transcript
*MGMT*: O-6-methylguanine-DNA methyltransferase
*MINT1*/*2*/*32*/*33*: Methylated-in-tumor (*MINT*) loci
*MIR10B*: MicroRNA 10b
*MIR124*-*1*/-*3*: MicroRNA MIR124 family
*MIR124*-*2HG*: MIR124-2 host gene
*MIR130B*: MicroRNA 130b
*MIR210*: MicroRNA 210
*MIR9*-*1*: MicroRNA 9-1
*MLH1*: mutL homolog 1
*MSLN*: Mesothelin
*MUC2*: Mucin 2, oligomeric mucus/gel-forming
*MYB*: MYB proto-oncogene, transcription factor
*MYOD1*: Myogenic differentiation 1
*NDN*: Necdin, MAGE family member
*NDRG4*: NDRG family member 4
*NEUROG1*: Neurogenin 1
*NEUROG3*: Neurogenin 3
*NPR3*: Natriuretic peptide receptor 3
*NPTX2*: Neuronal pentraxin 2
*PCDH17*: Protocadherin 17
*PDE4B*: Phosphodiesterase 4B
*PENK*: Proenkephalin
*PGK1*: Phosphoglycerate kinase 1
*PGR*: Progesterone receptor
*PITX2*: Paired-like homeodomain 2
*PLAU*: Plasminogen activator, urokinase
*PNMT*: Phenylethanolamine N-methyltransferase
*PRKCB*: Protein kinase C beta
*PRMT1*: Protein arginine methyltransferase 1
*PSCA*: Prostate stem cell antigen
*RARB*: Retinoic acid receptor beta
*RASSF1A*: Ras association domain family member 1
*RB1*: RB transcriptional corepressor 1
*REG1A*: Regenerating family member 1 alpha
*RPRM*: Reprimo, TP53 dependent G2 arrest mediator homolog
*S100A4*: S100 calcium binding protein A4
*SEPTIN9*: Septin 9
*SFN*: Stratifin
*SFRP1*: Secreted frizzled-related protein 1
*SFRP2*: Secreted frizzled-related protein 2
*SIM1*: SIM bHLH transcription factor 1
*SIM2*: SIM bHLH transcription factor 2
*SMARCA1*: SWI/SNF related, matrix associated, actin dependent regulator of chromatin, subfamily a, member 1
*SMOC2*: SPARC-related modular calcium binding 2
*SOCS1*: Suppressor of cytokine signaling 1
SOX3: SRY-box transcription factor 3
*SPARC*: Secreted protein acidic and cysteine rich
*SST*: Somatostatin
*ST14*: Suppression of tumorigenicity 14
*SYK*: Spleen-associated tyrosine kinase
*TAC1*: Tachykinin precursor 1
*TBX5*: T-box transcription factor 5
*TFAP2C*: Transcription factor AP-2 gamma
*TFF2*: Trefoil factor 2
*TFPI2*: Tissue factor pathway inhibitor 2
*THBS1*: Thrombospondin 1
*TIMP3*: TIMP metallopeptidase inhibitor 3
*TJP2*: Tight junction protein 2
*TP73*: Tumor protein p73
*TRADD*: TNFRSF1A associated via death domain
*TWIST1*: Twist family bHLH transcription factor 1
*UCHL1*: Ubiquitin C-terminal hydrolase L1
*VHL*: von Hippel-Lindau tumor suppressor
*VIM*: Vimentin
*VSTM2B*: V-set and transmembrane domain containing 2B
*WNT3*: Wnt family member 3
*WNT5A*: Wnt family member 5A
*WNT7A*: Wnt family member 7A
*ZBP*: Z-DNA Binding Protein
*ZBTB16*: Zinc finger and BTB domain containing 16
*ZNF415*: Zinc finger protein 415
